# Effect of community-based public health service on health-related quality of life among middle-aged and older adults with chronic diseases in China

**DOI:** 10.1186/s12889-024-19556-w

**Published:** 2024-07-30

**Authors:** Jingxian Wu, Danlei Chen, Cong Li, Yingwen Wang

**Affiliations:** School of Economics and Finance, Xi’an Jiaotong University, Xi’an, Shaanxi P.R. China

**Keywords:** Community-based public health service, Health-related quality of life, Chronic disease patients, Middle-aged and older Chinese adults

## Abstract

**Background:**

The growing prevalence of non-communicable chronic diseases poses a significant public health challenge globally, particularly impacting the well-being of aging populations. This study aims to assess the impact of community-based public health service (PHS) on the health-related quality of life (HRQoL) among middle-aged and older adults with chronic diseases in China.

**Methods:**

Utilizing data from the China Health and Retirement Longitudinal Study, we constructed a novel scale based on the 36-Item Short Form Health Survey (SF-36) to measure the HRQoL of middle-aged and older patients with hypertension and/or type-2 diabetes. Multivariate linear regression models with Instrument Variables and Propensity Score Matching techniques were applied to examine the effect of PHS on the HRQoL of identified chronic disease patients.

**Results:**

Among 8,403 hypertensive and/or diabetic patients, only 10.98% had received PHS. After adjusting for covariates, PHS exhibited a significant association with an elevated overall SF-36 score (β = 3.539, *p* < 0.001). Similar effects were observed in the physical and mental component summary scores, with increases of 1.982 (*p* < 0.001) and 5.095 (*p* < 0.001), respectively. Sensitive analysis affirmed the robustness of these findings. Heterogeneity analysis revealed significant HRQoL improvements among males, females, those aged 70 and older, patients with comorbidities, and urban residents, while the effect was less pronounced in the middle-aged, those without comorbidities, or rural dwellers.

**Conclusion:**

Community-based PHS has exerted a positive impact on both the physiological and psychological aspects of HRQoL among middle-aged and older chronic disease patients, with effects varying among individuals with different characteristics. Our findings advocate for enhancing the delivery and utilization of government-funded PHS, increasing health literacy, and promoting early prevention strategies for chronic diseases. Furthermore, targeted health management initiatives for patients with comorbidities and enhancements in the quality of community healthcare services, particularly in rural areas, are deemed necessary.

**Supplementary Information:**

The online version contains supplementary material available at 10.1186/s12889-024-19556-w.

## Introduction

The ongoing evolution of socioeconomic dynamics and lifestyle behaviors, coupled with the escalating challenges of an aging population, has led to a global increase in the morbidity and mortality rates of non-communicable chronic diseases, such as hypertension, diabetes, and coronary heart disease, over the past decades. This escalation poses a significant public health concern, profoundly impacting the well-being of populations worldwide [1], and China is no exception. By 2018, over 300 million people in China were affected by chronic diseases, with the middle-aged and elderly constituting more than 60% of this population [2]. Chronic diseases, characterized by their diversity, complexity, insidious onset, prolonged duration, and propensity for persistence and recurrence, are manageable rather than fully curable [3]. Evidence indicates that chronic disease patients often face dual challenges to their physical and psychological well-being, resulting in a relatively poor health-related quality of life (HRQoL) [4]. In China, for instance, chronic diseases contribute to over 70% of disability-adjusted life years lost and 88% of fatalities [1]. The escalating expenditure on chronic disease treatment has imposed a substantial economic burden on families, accounting for 80% of total health expenditure, thereby posing challenges to the economic sustainability of the nation’s healthcare system [5].

Community-based public health interventions, including family doctor services, routine physical or health examinations, chronic disease screening, and health education, have been recognized as cost-effective measures for preventing and managing chronic disease [6, 7]. In 2009, the Chinese government initiated a comprehensive healthcare reform plan, with a significant focus on providing equitable, accessible, and free essential public health service (PHS) to all citizens [8]. The primary target groups for essential PHS include community-dwelling adults aged 35 and above with hypertension or type-2 diabetes. According to the National Essential Public Health Guidelines, PHS programs for chronic disease management include chronic disease screening, regular face-to-face health monitoring, routine health examinations, and categorized interventions for personalized treatment and health guidance facilitated by community healthcare professionals [8]. Government subsidies for these PHS programs have increased from RMB 15 (USD 2.14) per capita in 2009 [8] to RMB 74 (USD 10.46) per capita in 2020 [9]. As the essential PHS programs continue to expand, the management of other chronic diseases, such as tuberculosis and severe mental illnesses, has also been included. These programs are expected to significantly advance the comprehensive management of chronic diseases, ultimately enhancing the health outcomes and quality of life for community-dwelling individuals grappling with chronic conditions [10].

HRQoL is as a multidimensional measure of well-being, encompassing physical functioning, mental health, and socially relevant roles perceived by individuals over time [11]. It provides a comprehensive assessment of a patient’s overall state of survival, making it a valuable tool for informing decision-makers [13] and shaping policy agendas [14]. Given the high morbidity and mortality rates associated with chronic diseases, researchers have increasingly focused on assessing both patients’ survival status and HRQoL [12, 13]. Numerous studies have measured the HRQoL among chronic disease patients using various scales, such as the 36-Item Short Form Health Survey (SF-36) [15], the 3 or 5 Level Version of European Quality of Life 5 Dimensions [16], and questionnaire-based scales [17]. Furthermore, research has explored factors influencing the HRQoL of chronic disease patients, including demographic and socioeconomic characteristics [13, 15, 18], medication adherence, lifestyle behaviors [19], and the accessibility of healthcare programs such as public health insurance schemes [20, 21].

While existing research demonstrates the positive impact of community-based PHS programs on the management and control of chronic diseases [21, 22], a significant gap persists regarding their impact on patients’ HRQoL. For instance, evidence from various countries, including the UK [23], USA [24, 25], and China [26–28], indicates that PHS initiatives such as chronic disease screening and engagement with family doctors effectively mitigate disease progression by enhancing medication adherence and promoting healthy lifestyles. However, the correlation between these PHS initiatives and HRQoL among chronic disease patients remains inadequately explored. To date, only two studies—one from Norway [29] and another from China [17]—have suggested that the family doctor system enhances the HRQoL of older patients dealing with polypharmacy or diabetes. Despite being a vital component of publicly funded PHS initiatives in China, there is a lack of evidence on evaluating the impact of community-based routine health examinations on the HRQoL of chronic disease patients. Only one study has examined the enhancement of HRQoL among non-chronically ill older adults through such a PHS initiative [30]. Therefore, further investigation is imperative to comprehensively assess the impact of these public health interventions on the HRQoL outcomes among older individuals affected by chronic diseases.

To address this knowledge gap, this study aims to evaluate the effect of community-based routine health examinations, a vital component of the government-funded PHS program, on the HRQoL among middle-aged and older adults with chronic disease in China. We seek to determine whether this PHS program improves the HRQoL of middle-aged and older patients with hypertension and/or type-2 diabetes, given the prevalence of these chronic conditions and their focus within China’s community-based PHS framework. Using data from the China Health and Retirement Longitudinal Study (CHARLS), which provides a nationally representative sample of adults aged 45 and older, we employed empirical methods to analyze the impact, considering potential variations across different demographic and clinical profiles. We hope that such analysis will provide crucial insights for guiding the formulation of community-based public health intervention strategies and promoting healthy aging in China.

## Methods

### Data

The data for this study were sourced from the third wave of the CHARLS, conducted between July and September 2015. CHARLS is a nationwide survey targeting individuals aged 45 years and older in China. Its data collection involves face-to-face interviews, physical examinations, and biological sample collection, providing a high-quality, nationally representative sample for scientific research on health, socioeconomic status, and quality of life as people age. The national baseline survey, conducted in 2011–2012, includes approximately 10,000 households and 17,500 individuals, encompassing 150 counties/districts and 450 villages/urban communities across 28 provinces in China. Participants are followed up every two years, with subsequent waves in 2013, 2015, and 2018. To ensure the sample’s representativeness, the CHARLS team employs a multistage stratified probability-proportionate-to-size sampling method. Using an innovative software package (CHARLS-Gis), comprehensive lists of all dwellings in each area were created, and households were randomly selected from these lists. Within each selected household, one individual aged 45 or older was randomly chosen as the main respondent, with their spouse automatically included in the sample. Households without residents aged 45 or older were excluded from the survey. To minimize error and ensure accuracy, each stage of sampling was computerized, and all interviews were conducted using computer-aided personal interview technology. Detailed information about the CHARLS sampling procedure and data quality management can be found elsewhere [31, 32]. When compared with Chinese national population census data, the CHARLS sample closely matched the national population in terms of age distribution, gender ratio, marital status, and urban-rural distribution [32], confirming its representativeness for the entire population.

Given that physical examinations and blood tests in the CHARLS are administered biennially (2011–2012 and 2015), this study utilizes data from the 2015 wave, which includes the most recent physical checkup and blood test information. The 2015 CHARLS survey, following the established sampling method, interviewed a total of 20,967 individuals from 12,235 households. The data used in this research was obtained from three channels within the 2015 CHARLS dataset: (1) structured questionnaire surveys conducted through face-to-face interviews within the selected households; (2) physical examinations performed on site by proficient interviewers using standardized equipment; and (3) laboratory blood tests conducted at designated local healthcare institutions subsequent to the interview. The questionnaire survey and blood-based biomarker sample collection study adhered to a protocol approved by the Ethical Review Committee (Institutional Review Board, IRB) of Peking University (IRB 00001052–11014 & 00001052–11015), with each participant receiving two duly signed copies of the informed consent form. Full details of the physical examination and blood collection process have been published previously [33].

As China’s essential PHS programs specifically target community-dwelling individuals aged 35 and above diagnosed with hypertension and type-2 diabetes [8], this study includes self-reported and objectively measured hypertensive and/or diabetic patients as the analytical sample. Participants without these chronic conditions were excluded from the 2015 CHARLS dataset. Data regarding self-reported hypertension and diabetes were collected through the household questionnaire survey, where individuals were asked, “Have you received a diagnosis of hypertension/diabetes from a doctor?” (yes = 1, no = 0). Blood pressure measurements were taken three times at 5-minute intervals during a single occasion using an electronic monitor (Omron model HEM-7112) as part of the physical examination administered by the CHARLS team. We derived the mean of the three blood pressure measurements as the actual blood pressure value. Hypertension was defined according to World Health Organization guidelines [34] as a systolic blood pressure ≥ 140 mm Hg and/or diastolic blood pressure ≥ 90 mm Hg, or current use of antihypertensive medication. Diabetes was identified based on glycated hemoglobin (HbA1c) levels, with HbA1c values ≥ 6.5% indicating diabetes [35]. Blood samples for HbA1c analysis were collected and processed according to standardized protocols. While HbA1c is not the most widely used screening test for diabetes, it is recommended as an alternative method in various surveys [35].

**Fig. 1:**
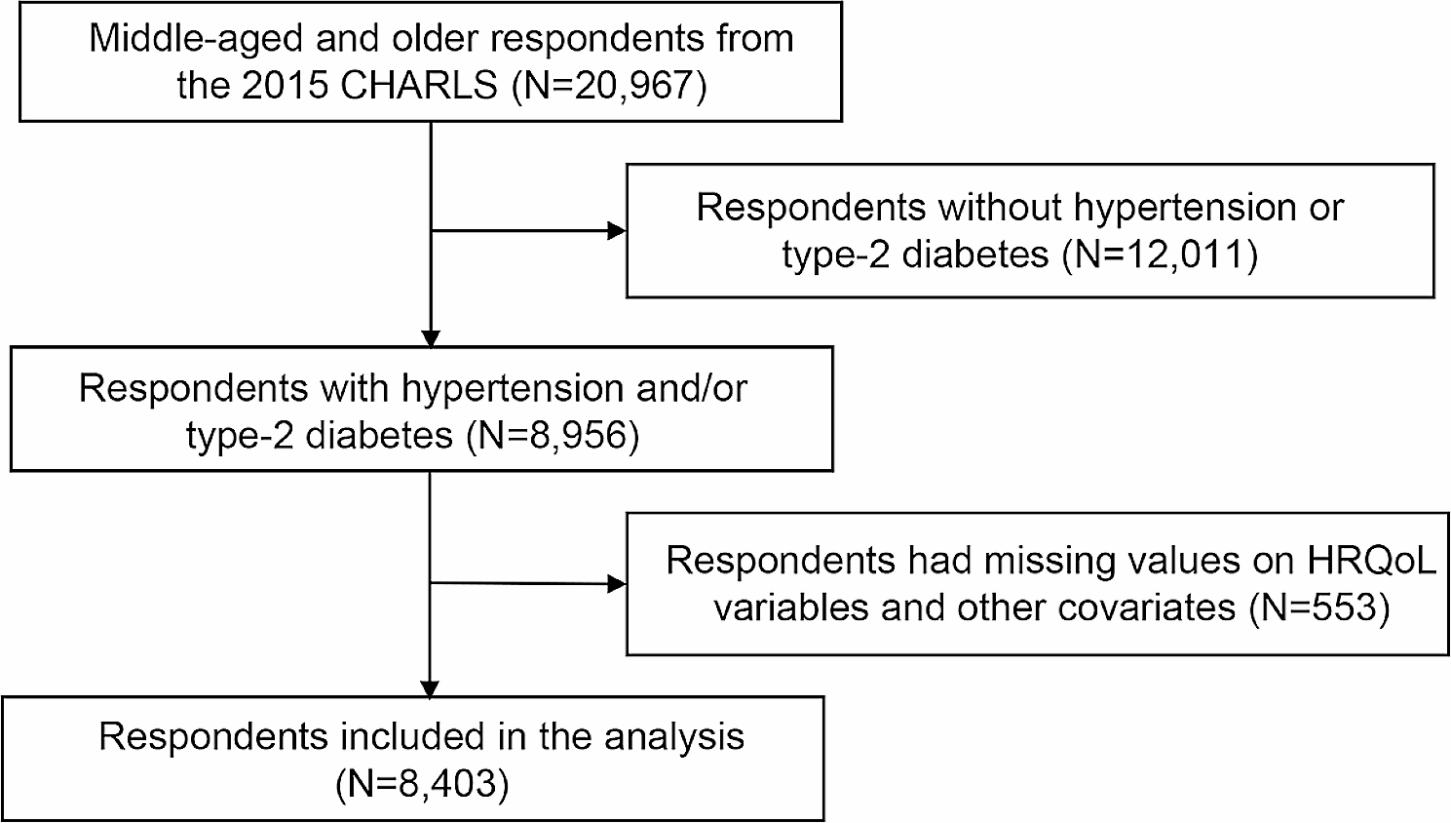
Sample inclusion process

The original 2015 CHARLS dataset comprised 20,967 respondents. Figure 1 illustrates the sample inclusion process. Initially, 12,011 individuals without self-reported or objectively measured hypertension and/or type-2 diabetes were excluded, reducing the sample to 8,956 respondents. Subsequently, cases with incomplete or missing data on HRQoL variables and covariates were removed. Following these data cleaning procedures, a final valid sample of 8,403 respondents with hypertension and/or diabetes was available for analysis.

### Measurement

#### Dependent variables

This study used a new scale constructed based on the SF-36 and CHARLS variables to measure the HRQoL among middle-aged and older adults with chronic disease. The SF-36, a frequently used self-administered screening tool, consists of 36 questions assessing eight health concepts defining quality of life [36]. While the CHARLS does not contain SF-36, it includes a wide range of health indicators on health status, physical functioning, mental health (depression), and cognitive capabilities. Following previous studies [30, 37, 38], we developed a new scale based on the CHARLS variables by selecting and adapting questionnaire items to assess the following eight dimensions of the original SF-36: physical function (PF), role–physical (RP), body pain (BP), general health (GH), vitality (VT), social functioning (SF), role–emotion (RE), and mental health (MH) [36]. Table 1 presents the selected variables in the CHARLS data for constructing the SF-36 scales.

**Table 1:** Corresponding variables in the CHARLS data for SF-36 scales

HRQoL (SF-36 Scales)		CHARLS variables
Physical Component Summary (PCS)	Physical functioning (PF)	Run 1 Km (DB001); Wald 1 Km(DB002); Walk 100 M (DB003); Difficulty with Getting up from Chair (DB004); Difficulty with Climbing Stairs (DB005); Difficulty with Kneeling (DB006); Difficulty with Extending Arms (DB007); Difficulty with Lifting (DB008); Difficulty with Picking up Coins (DB009)
	Role-physical (RP)	Difficulty with Household Chores (DB016); Difficulty with Preparing Meals (DB017); Difficulty with Shopping (DB018); Difficulty with Taking Medications (DB20)
	Bodily pain (BP)	Troubled With Any Body Pains (DA041); What Part Body Pain (DA042)
	General Health (GH)	Self-rated Health1 (DA001); Self-rated Health 2 (DA002)
Mental Component Summary (MCS)	Vitality (VT)	Sleep Restless (DC015);Could Not Get Going (DC018)
	Social functioning (SF)	Any Social Activities (DA056);Frequency of Social Activity (DA057)
	Role-emotional (RE)	Trouble with Concentrating (DC010); Feel Hard (DC012)
	Mental health (MH)	Usually Bothered (DC009); Felt Depressed (DC011); Felt Hopeful (DC013); Felt Fearful (DC014); Felt Happy (DC016); Felt Lonely (DC017)

Note: CHARLS, the China Health and Retirement Longitudinal Study. The corresponding item numbers from the household questionnaire of the 2015 CHARLS are provided in parentheses; see the official questionnaire for more details

We first recoded the values for the selected CHARLS variables so that higher item values indicated a poorer health state. The scores for each of the eight dimensions of the SF-36 were then calculated by adding up the category scores and converting the raw scores to a 0-to-100 scale. A higher score on this scale indicates a better quality of life for that dimension. Next, the scores for the eight subscales were aggregated into two summary scores, namely, the Physical Component Summary (PCS) and the Mental Component Summary (MCS) scores, based on the conceptual model of the SF-36: PCS includes PF, RP, BP, and GH, while MCS comprises VT, SF, RE, and MH [36, 39]. For samples with missing values in either PCS or MCS scores, imputation was performed by taking the mean of the available values. The overall SF-36 score was computed as the mean of the PCS and MCS scores.

The constructed SF-36 scale based on the CHARLS variables, although slightly different from other HRQoL questionnaires, has been deemed effective in assessing the health status of Chinese adults and the older population [30]. Except for VT (Cronbach’s $\:{\alpha\:}$ = 0.43) and RE (Cronbach’s $\:{\alpha\:}$ = 0.65), each dimension of the constructed SF-36 has an alpha value greater than 0.7, indicating acceptable internal consistency and good reliability. The comprehensive methodology for constructing this HRQoL scale, including detailed steps, reliability analysis, and validity assessment, is thoroughly documented in another publication [30].

#### Independent variable

This study defined the use of community-based PHS as whether a hypertensive or diabetic respondent received a government-subsidized health examination at community healthcare centers in the past two years. According to the Chinese National Essential Public Health Guidelines, community-based PHS programs for chronic disease management include chronic disease screening, regular face-to-face health monitoring, and categorized interventions for personalized treatment. Additionally, the program provides community-dwelling residents aged 35 years or older diagnosed with hypertension or diabetes with a government-subsidized comprehensive health examination once a year, which is available at any time throughout the year. This examination includes lifestyle and health status assessments, physical checkups (including measurements of blood pressure, height, weight, oral health, vision, waist circumference, and routine checks of the heart, lungs, and abdomen), auxiliary tests (including tumor markers, complete blood count, urinalysis, liver function tests, kidney function tests, blood glucose, and HbA1c), and targeted health guidance based on the examination results. In this study, information regarding the last health examination and payment source was retrieved from the questions “*When did you take the last health examination?*” (EC001) and “*Who paid for your last health examination?*” (EC002) in the CHARLS. Respondents who had a government-paid health examination in 2014 or 2015 were identified as PHS recipients. Those reporting health examinations paid by children, relatives, employers, insurance, themselves, or others were excluded to estimate the net effect of community-based PHS on HRQoL. These individuals were grouped with those who did not receive any health examination service, serving as the comparison group.

#### Control variables

Respondents’ demographic and socioeconomic characteristics, known potential factors influencing HRQoL, were included as control variables. These factors include age, gender, educational attainment, occupational status, marital status (i.e., living with a partner/spouse or not), presence of children in the same city or county (i.e., with a child living nearby or not), number of self-reported chronic diseases other than hypertension and diabetes, number of disabilities, household economic level (measured as household annual expenditure per capita, log-transformed), and health insurance participation status (i.e., having social health insurance [SHI] or not and having private health insurance [PHI] or not). To estimate the net effect of PHS on HRQoL, we included whether the respondents received non-government-subsidized health examinations as a covariate. Considering the significant disparity in regional socioeconomic development in China, we also controlled for respondents’ area of residence (rural = 0, urban = 1) and the level of regional socioeconomic development (measured as the GDP per capita of the sampled city where the respondents resided, log-transformed). We did not include medication adherence and health behaviors as control variables, as these behaviors may be influenced by the availability of routine health examinations [40]. Controlling for these variables could potentially obscure or alter the impact of such PHS on patients’ health outcomes.

### Empirical methods

Based on the descriptive statistical analysis of the sample characteristics mentioned above, we conducted regression analyses to examine the impact of community-based PHS on HRQoL of identified chronic disease patients. As the dependent variables (i.e., SF-36 scores) are numerical, multivariate linear regression models were employed. The baseline regression model is as follows:

1 $$\:{HRQoL}_{i}=\alpha\:+\beta\:{PHS}_{i}+\gamma\:{X}_{i}+{\epsilon\:}_{i}$$

In Eq. (1), $\:{HRQoL}_{i}$ represents the latent HRQoL of respondent *i*, $\:{PHS}_{i}$ is the explanatory variable representing the decision to use PHS by respondent *i*, $\:{X}_{i}$ is a column vector of covariates affecting the respondent’s HRQoL, and $\:{\epsilon\:}_{i}$ is the residual term. Clustering standard errors were applied within the community level due to potential correlations among respondents in the same community.

Three sensitivity analyses were conducted on the restricted sample, which excluded respondents who received non-government-subsidized health examination. Firstly, we re-conducted the multivariate linear regressions on the restricted sample. Secondly, we introduced instrumental variables (IVs) into the linear regression models because the accuracy of estimates regarding the relationship between community-based PHS and HRQoL may be compromised by the issue of endogeneity. For instance, respondents with heightened health awareness might be healthier, and consequently, more inclined to seek PHS, posing a risk of bias in our estimation. To enhance the reliability of our findings, we addressed this potential endogeneity by introducing IVs into our linear regression model. An ideal IV should be exogenous, strongly correlated with the treatment (i.e., the respondent’s decision to utilize PHS), and uncorrelated with both observed and unobserved characteristics related to the outcome (i.e., the respondent’s HRQoL). In this study, we selected two IVs to assess the impact of PHS on the HRQoL of chronic patients: whether the respondent’s partner/spouse utilized PHS (i.e., received government-subsidized health examinations) and the community-level utilization rate of PHS. The utilization rate of PHS for the *i-*th respondent ($\:{UR}_{i}$) in a specific community with *n* respondents is calculated as:

2 $$\:{UR}_{i}=\frac{{\sum\:}_{k=1}^{n}{PHS}_{k,k\ne\:i}-{PHS}_{i}}{n-1}$$

In Eq. (2), *k* denotes the *k-*th respondent in the community. The utilization rate variables are expected to be exogenous, not directly linked to the HRQoL of the respondents themselves. Moreover, they are anticipated to influence the respondents’ decision to use PHS due to the potential peer effect of health-related behaviors among individuals in the community [41]. To validate the exogeneity and validity of the IVs, we conducted a two-stage least squares (2SLS) regression, considering *P* values of Hansen J statistic and Cragg-Donald Wald F statistic.

Thirdly, considering that the receipt of community-based PHS may be influenced by the self-selective behavior of chronic patients, introducing a potential source of selection bias in our estimation [42], we employed propensity score matching (PSM) technique to address this issue. The PSM model, resembling multiple linear regressions, aims to mitigate biases arising from self-selection. It is crucial to note that unbiased estimation in multiple regressions relies on the accurate specification of the functional form; any deviation may introduce a bias, known as functional form misspecification (FFM). PSM, by contrast, diminishes the reliance on the functional form through matching, thereby relaxing the linearity assumption of the multiple regression models and mitigating the concerns associated with FFM [42]. In light of this, we opted to exclude respondents who had undergone non-government-funded health examinations and conducted an additional sensitivity analysis using the PSM method. The variables used for matching were consistent with the covariates in the preceding regression models.

All data analyses were performed using Stata 17.0, with a significance level set at $\:p<0.05$.

## Results

### Descriptive analysis

From the 2015 CHARLS dataset, we identified 8,403 respondents with hypertension and/or diabetes as our analytical sample, of whom only 923 (10.98%) had undergone community-based PHS. Table 2 provides descriptive statistics. The average overall SF-36 score was 57.575, with a slightly higher score for those who received PHS (mean: 58.551 vs. 57.454, *p* < 0.1). PHS recipients reported elevated PCS (mean: 55.125 vs. 54.985, *p* > 0.1) and MCS (mean: 61.978 vs. 59.923, *p* < 0.05) scores, indicating improved HRQoL compared to non-recipients. Regarding demographics, the average age of the analytical sample was 63.464 years, with PHS recipients being older (mean age: 66.955 vs. 63.033 years, *p* < 0.001) than non-recipients. PHS recipients also reported poorer health (mean number of other chronic diseases: 2.819 vs. 2.601, *p* < 0.001) but had higher educational attainment, were more likely to be unemployed/retired (51.90% vs. 44.57%, *p* < 0.001), and had a child living nearby (88.84% vs. 85.03%, *p* < 0.05). Although PHI recipients were more likely to live with a partner or spouse, this difference was not statistically significant (*p* > 0.1). Additionally, PHS users were more likely to be insured by SHI (92.09% vs. 88.04%, *p* < 0.001), to reside in economically developed cities (mean GDP per capita: USD 8,623.89 vs. USD 7,211.75), and to live in urban areas (45.50% vs. 39.76%, *p* < 0.001).

**Table 2:** Descriptive statistics

Variable	Total (*N* = 8,403)	PHS recipients (*N* = 923, 10.98%)	PHS non-recipients (*N* = 7,480, 89.02%)	*P* value
*Dependent variable*				
SF-36	57.575 (15.253)	58.551 (15.346)	57.454 (16.142)	0.050
PCS	55.000 (12.475)	55.125 (11.964)	54.985 (12.538)	0.748
MCS	60.149 (25.593)	61.978 (24.774)	59.923 (25.684)	0.021
*Control variable*				
Age (years)	63.464 (9.730)	66.955 (8.538)	63.033 (9.781)	< 0.001
Gender (%)				0.998
Male	47.23	47.23	47.23	
Female	52.77	52.77	52.77	
Education (%)				0.001
Illiterate	28.85	28.54	31.31	
Primary school	39.95	39.53	43.34	
Middle school	19.80	20.23	16.36	
High school and above	11.40	11.70	8.99	
Occupation (%)				< 0.001
Without employment or retired	45.38	51.90	44.57	
Agricultural work	32.89	32.29	32.97	
Non-agricultural work	21.73	15.82	22.46	
Living with a partner/spouse (%)				0.249
Yes	78.15	79.63	77.97	
No	21.85	20.37	22.03	
A child living nearby (%)				0.002
Yes	85.45	88.84	85.03	
No	14.55	11.16	14.97	
Number of other chronic diseases	2.625 (1.750)	2.819 (1.814)	2.601 (1.740)	< 0.001
Number of disabilities	0.214 (0.529)	0.232 (0.567)	0.211 (0.525)	0.264
SHI (%)				< 0.001
Yes	88.49	92.09	88.04	
No	11.51	7.91	11.96	
PHI (%)				0.403
Yes	2.11	1.73	2.15	
No	97.89	98.27	97.85	
Household expenditure per capital (in USD)	2,231.603 (2,783.803)	2,237.667 (2,390.959)	2,230.855 (2,828.6396)	0.944
City GDP per capital (in USD)	7,366.870 (4,132.628)	8,623.897 (4,685.976)	7,211.758 (4,032.402)	< 0.001
Area of residence (%)				0.001
Urban	40.39	45.50	39.76	
Rural	59.61	54.50	60.24	
Non-government-funded health examination (%)				< 0.001
Yes	36.82	0.00	41.36	
No	63.18	100.00	58.64	
*Instrumental variable*				
Respondents’ partner/spouse utilized PHS (%)				< 0.001
Yes	8.51	43.23	4.22	
No	91.49	56.77	95.78	
Community-level PHS utilization rate (%)	8.90 (0.09)	15.97 (0.31)	8.03 (0.09)	< 0.001

Note: SF-36, 36-Item Short Form Health Survey; PCS, physical component summary score; MCS, mental component summary score; SHI, social health insurance; PHI, private health insurance; GDP, Gross Domestic Product; PHS, public health service. Results are presented as mean (standard deviation) unless otherwise specified

### Baseline regressions

The impact of community-based PHS on HRQoL in middle-aged and older adults with chronic diseases is detailed in Table 3, reflecting the baseline multivariate linear regressions. After covariate adjustments, PHS demonstrated a significant association with an increased overall SF-36 score (β = 3.539, *p* < 0.001) among this demographic. A similar effect was observed in the SF-36 aggregate scores, with PHS recipients experiencing elevated PCS and MCS scores by 1.982 (*p* < 0.001) and 5.095 (*p* < 0.001), respectively. These outcomes underscore a noteworthy enhancement in both physiological and psychological quality of life following the utilization of PHS by chronic patients. Further subgroup analysis for respondents with hypertension and diabetes indicated a consistent positive effect. Multiple linear models revealed that PHS led to a substantial rise in SF-36 scores—overall (β-hypertension = 3.668, *p* < 0.001; β-diabetes = 4.927, *p* < 0.001), PCS (β-hypertension = 2.283, *p* < 0.001; β-diabetes = 2.773, *p* < 0.001), and MCS (β-hypertension = 5.054, *p* < 0.001; β-diabetes = 7.081, *p* < 0.001) in both groups. This aligns with the overall sample results, indicating the positive impact of PHS on HRQoL for middle-aged and older individuals with hypertension and diabetes.

**Table 3:** Effect of community-based PHS on HRQoL among chronic disease patients

Variables	Patients with hypertension and/or diabetes			Patients with hypertension			Patients with diabetes		
	SF-36	PCS	MCS	SF-36	PCS	MCS	SF-36	PCS	MCS
PHS	3.539^***^	1.982^***^	5.095^***^	3.668^***^	2.283^***^	5.054^***^	4.927^***^	2.773^***^	7.081^***^
	(0.539)	(0.428)	(0.873)	(0.577)	(0.466)	(0.925)	(0.966)	(0.729)	(1.570)
Age	-0.210^***^	-0.213^***^	-0.207^***^	-0.220^***^	-0.219^***^	-0.221^***^	-0.148^***^	-0.187^***^	-0.110
	(0.023)	(0.016)	(0.038)	(0.024)	(0.018)	(0.041)	(0.039)	(0.030)	(0.064)
Female	-1.354^***^	0.139	-2.847^***^	-1.228^**^	0.334	-2.791^***^	-0.932	0.052	-1.914
	(0.381)	(0.279)	(0.627)	(0.408)	(0.307)	(0.667)	(0.640)	(0.480)	(1.062)
Education (reference = illiteracy)									
Primary school	2.364^***^	0.898^*^	3.830^***^	2.401^***^	0.908^*^	3.895^***^	2.363^**^	1.154	3.573^**^
	(0.430)	(0.369)	(0.697)	(0.458)	(0.391)	(0.745)	(0.735)	(0.649)	(1.193)
Middle school	3.935^***^	0.929^*^	6.942^***^	4.011^***^	0.858	7.164^***^	3.967^***^	1.264	6.671^***^
	(0.511)	(0.406)	(0.830)	(0.560)	(0.442)	(0.907)	(0.946)	(0.790)	(1.495)
High school and above	6.562^***^	2.141^***^	10.980^***^	6.159^***^	1.976^***^	10.340^***^	7.813^***^	3.268^***^	12.360^***^
	(0.614)	(0.490)	(1.036)	(0.654)	(0.521)	(1.116)	(1.001)	(0.849)	(1.624)
Occupation (reference = without employment or retired)									
Agricultural work	3.921^***^	5.678^***^	2.164^**^	4.038^***^	5.760^***^	2.316^**^	3.669^***^	6.172^***^	1.166
	(0.408)	(0.314)	(0.693)	(0.431)	(0.332)	(0.728)	(0.712)	(0.597)	(1.199)
Non-agricultural work	4.100^***^	6.130^***^	2.071^*^	4.330^***^	6.361^***^	2.301^**^	3.936^***^	6.045^***^	1.828
	(0.475)	(0.342)	(0.804)	(0.518)	(0.368)	(0.876)	(0.794)	(0.567)	(1.377)
Living with a partner/spouse	1.819^***^	0.031	3.607^***^	1.839^***^	0.121	3.556^***^	1.319	-1.061	3.699^**^
	(0.434)	(0.342)	(0.724)	(0.463)	(0.370)	(0.758)	(0.749)	(0.573)	(1.255)
A child living nearby	0.291	-0.461	1.042	0.309	-0.521	1.140	0.099	-0.244	0.441
	(0.445)	(0.318)	(0.801)	(0.488)	(0.352)	(0.851)	(0.742)	(0.586)	(1.333)
Number of other chronic diseases	-1.885^***^	-1.156^***^	-2.613^***^	-1.826^***^	-1.153^***^	-2.498^***^	-1.916^***^	-1.007^***^	-2.826^***^
	(0.105)	(0.081)	(0.171)	(0.110)	(0.084)	(0.181)	(0.155)	(0.133)	(0.247)
Number of disabilities	-5.516^***^	-3.913^***^	-7.119^***^	-5.759^***^	-4.111^***^	-7.408^***^	-5.502^***^	-3.405^***^	-7.598^***^
	(0.366)	(0.330)	(0.558)	(0.396)	(0.346)	(0.605)	(0.576)	(0.527)	(0.899)
SHI	1.806^***^	1.086^**^	2.526^**^	1.676^**^	0.834	2.518^*^	2.746^**^	2.077^**^	3.415^*^
	(0.537)	(0.418)	(0.912)	(0.576)	(0.451)	(0.980)	(0.939)	(0.744)	(1.592)
PHI	2.304^*^	0.535	4.073*	2.021	0.010	4.031^*^	3.564	1.901	5.2228
	(0.995)	(0.704)	(1.713)	(1.110)	(0.801)	(1.874)	(1.822)	(1.381)	(2.919)
Log household expenditure per capital	0.089	0.148	0.030	0.058	0.142	-0.026	0.163	-0.123	0.449
	(0.206)	(0.156)	(0.351)	(0.222)	(0.167)	(0.377)	(0.346)	(0.272)	(0.557)
Urban	2.561^***^	1.620^***^	3.502^***^	2.531^***^	1.577^***^	3.485^***^	2.060^**^	1.227^*^	2.894^**^
	(0.423)	(0.345)	(0.696)	(0.438)	(0.357)	(0.722)	(0.673)	(0.599)	(1.062)
Log GDP per capital	1.594^***^	0.845^**^	2.342^***^	1.747^***^	0.887^**^	2.606^***^	1.534^**^	0.788	2.279^*^
	(0.409)	(0.304)	(0.706)	(0.415)	(0.309)	(0.730)	(0.567)	(0.500)	(0.902)
Non-government-subsidized health examination	2.612^***^	1.042^***^	4.182^***^	2.733^***^	1.269^***^	4.197^***^	2.439^***^	1.410^**^	3.467^***^
	(0.328)	(0.279)	(0.530)	(0.364)	(0.312)	(0.583)	(0.576)	(0.508)	(0.924)
Constant	54.150^***^	57.860^***^	50.430^***^	53.295^***^	57.704^***^	48.886^***^	50.140^***^	57.520^***^	42.759^***^
	(4.024)	(2.884)	(7.138)	(4.093)	(2.943)	(7.350)	(5.959)	(5.005)	(9.841)
Observations	8,403	8,403	8,403	7,425	7,425	7,425	2,540	2,540	2,540
R-squared	0.228	0.219	0.157	0.230	0.223	0.158	0.229	0.199	0.170
F statistic	111.28^***^	108.77^***^	70.14^***^	106.53^***^	106.92^***^	67.01^***^	35.85^***^	27.31^***^	30.05^***^

Note: PHS, public health service; HRQoL, health-related quality of life; SF-36, 36-Item Short Form Health Survey; PCS, physical component summary score; MCS, mental component summary score; SHI, social health insurance; PHI, private health insurance; GDP, Gross Domestic Product. Cluster-robust standard errors in parentheses. ^*^p < 0.05, ^**^p < 0.01, ^***^p < 0.001

### Sensitive analysis

The aforementioned empirical findings demonstrated the positive impact of community-based PHS on the HRQoL of middle-aged and older adults with chronic diseases. To strengthen the reliability of the results, we conducted three sensitivity analyses on the restricted sample, which excluded respondents who received non-government-subsidized health examination. Table 4 presents the estimated adjusted coefficients of community-based PHS on HRQoL using multivariate linear regressions. The significantly positive coefficients of PHS demonstrate a beneficial effect on patients’ HRQoL, particularly on the mental component, which is consistent with the results of the baseline regressions.

The other two sensitivity analyses were also conducted on the restricted sample, addressing potential endogeneity concerns through the IVs and PSM methods. On the one hand, we mitigated endogeneity concerns using the IV method. As previously mentioned, we employed the utilization of PHS by respondents’ partners/spouses and the community-level utilization rate of PHS as instruments. Overidentification test results validated the instruments, confirming their lack of correlation with the error term. Additionally, the weak identification test proved the strength of the IVs [43]. Table 5 displays the effects of PHS on HRQoL using the IV model, revealing positive and statistically significant adjusted coefficients, particularly on MCS scores. These findings support the finding that community-based PHS positively influences the HRQoL in hypertensive and diabetic patients.

On the other hand, we estimated propensity scores via a logit model and employed matching through caliper method and nearest-neighbor method within a caliper. In conducting the nearest-neighbor matching, given the limited sample size in the treatment group, we opted for a one-to-four matching approach to mitigate sample loss, employing a caliper set at 0.25 times the standard deviation of the propensity score [44] (i.e., caliper = 0.015). Tables A1 to A6 in Additional file present the control variable test results before and after matching, indicating *p* values > 0.1 for all matched variables and nonsignificant differences in covariates between treatment and control groups post-matching, validating the parallel hypothesis. Figures A1 to A6 in Supplementary Material 1 show that the propensity scores of most sample were within the common value range; and meanwhile Figures A7 to A12 demonstrate that the kernel density distributions between treatment and control groups tended to coincide after matching, satisfying the common support hypothesis. Consequently, the matching process proves effective, enhancing the comparability of the matched samples. The results of multivariate linear regression on the matched samples (see Table 6) reveal that the adjusted regression coefficients of the PHS on SF-36 scores remain positive and statistically significant. This affirms the validity of baseline regression results after alleviating selection bias, reinforcing that community-based PHS improved the HRQoL for chronic patients.

**Table 4:** Adjusted coefficients of community-based PHS on HRQoL among chronic disease patients using multivariate linear regression

Variable	Patients with hypertension and/or diabetes			Patients with hypertension			Patients with diabetes		
	SF-36	PCS	MCS	SF-36	PCS	MCS	SF-36	PCS	MCS
PHS	3.665***	2.140***	5.189***	3.752***	2.452***	5.053***	5.179***	3.072***	7.286***
	(0.559)	(0.448)	(0.897)	(0.601)	(0.486)	(0.957)	(1.006)	(0.782)	(1.608)
Observations	5,309	5,309	5,309	4,715	4,715	4,715	1,491	1,491	1,491
R-squared	0.215	0.218	0.143	0.214	0.223	0.139	0.218	0.200	0.161
F statistic	71.46***	67.48***	47.61***	66.35***	65.03***	43.90***	21.77***	16.25***	18.26***

Note: PHS, public health service; HRQoL, health-related quality of life; SF-36, 36-Item Short Form Health Survey; PCS, physical component summary score; MCS, mental component summary score. Cluster-robust standard errors in parentheses. ^*^p < 0.05, ^**^p < 0.01, ^***^p < 0.001. The regression coefficients presented in this table have been adjusted for the aforementioned control variables

**Table 5:** Adjusted coefficients of community-based PHS on HRQoL among chronic disease patients using the IV method

Variable	Patients with hypertension and/or diabetes			Patients with hypertension			Patients with diabetes		
	SF-36	PCS	MCS	SF-36	PCS	MCS	SF-36	PCS	MCS
PHS	4.468***	3.376***	5.560**	4.480***	3.620***	5.340**	6.196***	3.795**	8.597**
	(1.149)	(0.844)	(1.965)	(1.226)	(0.917)	(2.070)	(1.756)	(1.338)	(2.807)
Observations	5,309	5,309	5,309	4,715	4,715	4,715	1,491	1,491	1,491
R-squared	0.215	0.217	0.143	0.214	0.222	0.139	0.218	0.200	0.161
F statistic	71.59***	66.33***	47.05***	66.11***	64.29***	43.19***	21.66***	16.22***	17.84***
*P* value of overidentification test using 2SLS	0.699	0.403	0.957	0.681	0.439	0.926	0.514	0.427	0.709
Cragg-Donald Wald F statistic	968.972	968.972	968.972	836.155	836.155	836.155	281.714	281.714	281.714
Stock-Yogo weak identification test critical values: 10% maximal IV size	19.93	19.93	19.93	19.93	19.93	19.93	19.93	19.93	19.93

Note: HRQoL, health-related quality of life; PHS, public health service; SF-36, 36-Item Short Form Health Survey; PCS, physical component summary score; MCS, mental component summary score; 2SLS, Two-Stage Least Squares; IV, instrument variable. Cluster-robust standard errors in parentheses. ^*^p < 0.05, ^**^p < 0.01, ^***^p < 0.001. The regression coefficients presented in this table have been adjusted for the aforementioned control variables

**Table 6:** Adjusted coefficients of community-based PHS on HRQoL among chronic disease patients using propensity score matched samples

Matching method	Patients with hypertension and/or diabetes			Patients with hypertension			Patients with diabetes		
	SF-36	PCS	MCS	SF-36	PCS	MCS	SF-36	PCS	MCS
Caliper matching	3.610***	2.102***	5.119***	3.712***	2.412***	5.012***	5.063***	2.943***	7.183***
	(0.558)	(0.445)	(0.899)	(0.600)	(0.485)	(0.959)	(1.010)	(0.783)	(1.621)
Nearest-neighbor matching within caliper	4.069***	2.285***	5.852***	3.963***	2.375***	5.551***	5.114***	2.738***	7.490***
	(0.601)	(0.488)	(0.951)	(0.624)	(0.519)	(1.005)	(1.061)	(0.835)	(1.715)

Note: HRQoL, health-related quality of life; PHS, public health service; SF-36, 36-Item Short Form Health Survey; PCS, physical component summary score; MCS, mental component summary score. Cluster-robust standard errors in parentheses. ^*^p < 0.05, ^**^p < 0.01, ^***^p < 0.001. The regression coefficients presented in this table have been adjusted for the aforementioned control variables

### Heterogeneity analysis

Examining the effects of PHS on middle-aged and older chronic patients with diverse characteristics is crucial for informing future policies and research initiatives. Recognizing the disparities in HRQoL based on age, gender, comorbidity, and urban-rural distinctions in China [13], our investigation explored the heterogeneous impact of community-based PHS among middle-aged and older chronic patients. We categorized the total sample by age, gender, presence of other comorbidities, and area of residence (urban or rural) to comprehensively understand the PHS program’s impact. Grouped regressions, based on a multivariate linear regression model (see Table 7), reveal substantial heterogeneity in the impact of PHS on the HRQoL of middle-aged and older chronic disease patients in urban and rural areas, as well as across different age groups and comorbidity statuses.

Specifically, PHS had shown a significant positive impact on HRQoL among both female and male chronic disease patients. However, this effect varies according to age. Among hypertensive and diabetic patients aged 70 and older, there was a noticeable increase in SF-36 scores after receiving PHS, whereas the effect was less pronounced in middle-aged patients (aged 45–59). Analysis of the SF-36 component summaries revealed that the beneficial effect of PHS on older hypertensive and diabetic patients was predominantly seen in an increased MCS score. These findings indicated significant improvements in HRQoL among older adults with chronic diseases due to PHS, thereby promoting healthy aging in China. In terms of comorbidity status, PHS was found to be associated with significant increases in SF-36 scores only among hypertensive and diabetic patients with comorbidities. This suggested that PHS enhanced the HRQoL of middle-aged and older adults with comorbidities, while its effect is less pronounced in individuals solely afflicted with hypertension and/or diabetes. Furthermore, when comparing the impact of PHS on HRQoL between urban and rural patients, significant improvements in SF-36, PCS, and MCS scores were observed among urban residents receiving PHS. However, the effect on rural residents, particularly diabetic patients, was less conspicuous.

**Table 7:** Adjusted coefficients of community-based PHS on HRQoL among chronic disease patients of different characteristics

Subgroups		Patients with hypertension and/or diabetes			Patients with hypertension			Patients with diabetes		
		SF-36	PCS	MCS	SF-36	PCS	MCS	SF-36	PCS	MCS
Age	45–59 years old	2.411^*^	0.764	4.057^*^	2.264^*^	1.013	3.516	2.364	0.591	4.138
		(0.939)	(0.760)	(1.676)	(1.027)	(0.825)	(1.820)	(1.452)	(1.204)	(2.621)
	60–69 years old	2.154^**^	0.747	3.561^**^	1.999^*^	0.781	3.217^*^	4.760^**^	2.445^*^	7.075^**^
		(0.769)	(0.602)	(1.316)	(0.802)	(0.645)	(1.371)	(1.466)	(1.061)	(2.461)
	≥ 70 years old	4.123^***^	3.184^***^	5.062^**^	4.575^***^	3.684^***^	5.467^**^	6.739^**^	5.199^**^	8.279^*^
		(1.062)	(0.819)	(1.760)	(1.097)	(0.869)	(1.793)	(2.072)	(1.655)	(3.263)
Gender	Male	3.867^***^	2.082^***^	5.652^***^	4.045^***^	2.383^***^	5.707^***^	6.067^***^	3.553^**^	8.580^**^
		(0.764)	(0.592)	(1.240)	(0.799)	(0.636)	(1.287)	(1.529)	(1.091)	(2.546)
	Female	3.209^***^	1.866^**^	4.552^***^	3.332^***^	2.222^***^	4.442^**^	4.337^***^	2.249^*^	6.424^**^
		(0.692)	(0.570)	(1.187)	(0.748)	(0.628)	(1.276)	(1.189)	(0.972)	(1.951)
Comorbidity status	With	3.527^***^	2.010^***^	5.043^***^	3.702^***^	2.290^***^	5.115^***^	4.899^***^	3.092^***^	6.706^***^
		(0.568)	(0.448)	(0.916)	(0.606)	(0.485)	(0.973)	(1.006)	(0.764)	(1.632)
	Without	3.377	1.539	5.215	2.620	1.881	3.360	6.231^*^	-1.847	14.309^**^
		(1.795)	(1.237)	(2.964)	(2.050)	(1.400)	(3.320)	(2.672)	(2.176)	(4.866)
Area of residence	Urban	4.667^***^	2.693^***^	6.640^***^	5.165^***^	3.354^***^	6.976^***^	7.214^***^	3.650^**^	10.778^***^
		(0.856)	(0.635)	(1.333)	(0.925)	(0.693)	(1.411)	(1.440)	(1.065)	(2.284)
	Rural	2.648^***^	1.421^*^	3.875^**^	2.472^**^	1.424*	3.520^**^	3.022^*^	2.139^*^	3.906
		(0.701)	(0.586)	(1.168)	(0.744)	(0.635)	(1.238)	(1.295)	(1.027)	(2.144)

Note: HRQoL, health-related quality of life; PHS, public health service; SF-36, 36-Item Short Form Health Survey; PCS, physical component summary score; MCS, mental component summary score. Cluster-robust standard errors in parentheses. ^*^p < 0.05, ^**^p < 0.01, ^***^p < 0.001. The regression coefficients presented in this table have been adjusted for the aforementioned control variables

## Discussion

### Main findings

Based on nationally representative survey data from the 2015 wave of the CHARLS, this study developed a novel SF-36 scale and employed multivariate linear regressions with IVs and PSM to examine the impact of community-based PHS on HRQoL among middle-aged and older adults with chronic diseases in China. Focusing on hypertensive and diabetic patients as the analytical sample, our results reveal that the receiving government-funded health examinations is associated with significant increases in the SF-36 scores for this population. In China, the government-subsided health examination service for community-dwelling chronic disease patients, as a critical component of community-based PHS framework, includes not only comprehensive physical checkups but also targeted health guidance. This service is accessible at any time throughout the year. In our sample, the average time gap between chronic disease patients being surveyed (when their health status and HRQoL were measured) and receiving PHS was about 7.8 months. This temporal gap allows us to attribute patients’ improvements in HRQoL to the recipient of PHS, despite using a cross-sectional dataset. This finding aligns with prior research that highlights the role of routine health checks and publicly-funded PHS in aiding chronic disease management and enhancing overall quality of life through increased awareness of current disease status, promotion of medication adherence, and positive self-management of health-related behaviors [21, 45]. The favorable effect of PHS on HRQoL is also anticipated to contribute to the reduction of escalating healthcare costs related to chronic disease treatment, thereby emphasizing the efficacy of government investments in public health.

Upon dividing the constructed SF-36 scale into physical and mental health subscales, this study substantiated significant enhancements in both PCS and MCS scores associated with receiving government-funded routine health examinations. This finding indicates that community-based PHS positively influences both the physiological and psychological well-being of hypertensive and diabetic patients. Notably, the impact on the mental well-being of chronic patients surpassed that on their physical well-being, as evidenced by higher adjusted coefficients of PHS with MCS scores compared to PCS scores. This outcome diverges from prior research focusing on older adults without chronic diseases [30], which suggested a more substantial impact of health management programs on physical rather than psychological well-being. A plausible explanation for this disparity is that individuals with chronic diseases typically face heightened risks of experiencing poorer mental health. Previous research consistently reveals an elevated likelihood of mental health disorders, such as anxiety and depression, among individuals with chronic diseases compared to the general population [46]. Moreover, depression, in comparison to an increased number of physical chronic conditions, exerts a more pronounced effect on diminishing HRQoL [37, 47]. The challenges associated with managing a chronic illness, coping with its symptoms, and the ensuing impact on daily functioning contribute to psychological distress, exacerbating physical symptoms and diminishing overall quality of life [48]. The provision of government-funded health examinations can enhance disease awareness and treatment compliance among patients with chronic diseases, offering valuable support in managing anxiety levels. Additionally, professional health guidance obtained after comprehensive physical check-ups serves as a motivating factor for adopting healthier behaviors, such as engaging in physical exercise. These behaviors possess a social dimension and have the potential to alleviate psychological stress among patients with chronic diseases, fostering a sense of vitality [49]. Consequently, improvements in the psychological aspects of quality of life may manifest in a relatively short period. This aligns with prior research, which suggests that psychological well-being may be closely tied to a high-quality life, potentially surpassing the influence of good physical health and favorable socioeconomic circumstances [50]. Conversely, the impact of PHS on physiological aspects of quality of life is not directly influential, as these aspects often necessitate medication or specific medical interventions.

Segmenting the samples based on their characteristics, our study contributed a comprehensive understanding of the heterogeneity in the effect of government-funded PHS on HRQoL. Firstly, our findings revealed a significant promotion effect of community-based health examinations on the HRQoL of hypertensive and diabetic patients aged 70 and older. However, this impact is not evident among middle-aged adults (i.e., those aged 45 to 59 years old). Prior research indicated a gradual decline in physical conditions and physiological functions among older adults as chronic diseases progress with age, exerting an increasing impact on health [51]. Older adults, in comparison to their middle-aged and young-old counterparts, possess lower digital literacy rates and may be less adept at utilizing emerging channels, such as the internet, to access chronic disease management advice [52]. Instead, they are more inclined to rely on traditional healthcare resources, including community health screenings and assessments, to receive health recommendations. Consequently, older adults are more amenable to benefiting from interventions targeting chronic diseases, such as community-based health examinations. In contrast, middle-aged adults typically contend with substantial pressures stemming from work and family life, limiting their available time and financial resources. Even when provided with recommendations for community-based chronic disease management programs, such as engaging in physical exercise, quitting smoking and alcohol, and routine blood pressure/sugar monitoring, middle-aged adults may struggle to fully comply with these recommendations. This limitation results in a constrained facilitation of their HRQoL by PHS. The intricate balance of responsibilities faced by middle-aged adults underscores the challenges they encounter in adopting and sustaining health-promoting behaviors, highlighting the need for tailored interventions that accommodate the unique circumstances of this demographic.

Secondly, our study brought to light a noteworthy observation: community-based PHS significantly enhance the HRQoL for hypertensive and diabetic patients with comorbidities, while such promotion effect was not that significant for those without comorbidities. This discrepancy may be attributed to poor health literacy among middle-aged and older residents in China, the majority of whom have only received primary education. This educational landscape contributes to a lack of emphasis on early management of chronic diseases [53]. For patients with only hypertension or diabetes, their physical discomfort may not be as pronounced, and their motivation to improve health behaviors may lack proactive initiative. It is not until the onset of comorbidities or complications that they begin to prioritize self-management of chronic conditions [53]. In contrast, patients with multiple chronic diseases need to manage several conditions concurrently, making them more sensitive to their health status and likely to undergo more medical and health education. Consequently, they possess deeper insights into the progression and hazards of chronic diseases, demonstrating greater attention to and understanding of how to manage and control these conditions to enhance their HRQoL. Routine health examinations play a pivotal role in providing comprehensive health assessments and intervention recommendations. These examinations facilitate multi-morbid patients in overall health management, encouraging them to become more actively engaged in self-management. This involvement includes complying with medical advice, undergoing regular check-ups and monitoring, and placing greater emphasis on dietary, exercise, and lifestyle modifications to alleviate the impact of diseases. These proactive measures contribute to the improvement of their HRQoL, emphasizing the crucial role of community-based interventions in fostering holistic health management, particularly for those with multiple chronic conditions.

Thirdly, this study indicated that community-based PHS proves to be more effective for urban patients with chronic diseases than for their rural counterparts. A possible interpretation of this finding revolves around the unequal distribution of healthcare resources between rural and urban areas, exacerbating the poor quality of community healthcare services in rural regions. Compared to urban areas, rural counterparts grapple with a scarcity of healthcare resources, encompassing insufficient government funding, limited medical equipment, and medications. The longstanding challenge of a shortage of qualified health workers in rural primary care centers further impedes the enhancement of healthcare quality [54], thereby hindering the effectiveness of community-based PHS for rural residents. Even with the provision of free health examination services, rural patients may encounter difficulties in accessing subsequent treatment and management, thereby constraining the promotional impact of PHS on their HRQoL. Additionally, rural residents often face challenges in accessing health information and medical knowledge due to lower educational attainment, health literacy, and restricted channels of access. This situation results in low awareness and willingness among rural residents to engage in health and disease self-management, ultimately discouraging them from benefiting fully from PHS. The findings underscore the imperative of advancing chronic disease management and equalizing access to PHS among the rural population in China, emphasizing the need for targeted interventions and resource allocation to bridge the existing healthcare disparities between urban and rural areas.

Last but not least, despite validating the effectiveness of community-based PHS on the HRQoL of chronic disease patients, a mere fraction of middle-aged and older individuals with chronic conditions (less than 11% in 2015) availed themselves of this service. The low utilization of PHS among chronic disease patients lies partially in their weak health consciousness. Typically, patients only become attentive to the self-management of chronic conditions when symptoms significantly impact their daily lives. Particularly, those residing in rural areas lack adequate knowledge and self-management instructions for chronic diseases [55]. This aligns with previous studies asserting that low literacy poses a significant obstacle to the effective implementation of public health interventions, including health education [10]. Additionally, the lack of trust among community-dwelling residents in the quality of primary healthcare services contributes to their reluctance to seek health management or medical treatment at community healthcare facilities. The shortage of healthcare workforce and medical equipment further hampers the delivery of preventive services, particularly in remote rural areas [10, 22, 56]. These challenges underscore the critical need for targeted health education, improved accessibility, and enhanced community trust-building initiatives to promote the utilization of PHS among individuals with chronic diseases, particularly in underserved rural regions.

### Limitations

Several limitations should be acknowledged in this study. Firstly, we utilized data from the 2015 wave of the CHARLS to investigate the impact of community-based PHS on the HRQoL of middle-aged and older patients with chronic diseases. While the study results possess a certain level of national representativeness, it is crucial to note that the latest blood test data were unavailable, preventing the presentation of the most up-to-date information regarding the effectiveness of community public health services. Additionally, the cross-sectional design of the current study limits the assessment of the long-term effects of PHS, which can be addressed when long-term follow-up data become available. Secondly, as individuals age, cognitive function may decline, introducing the possibility of biased estimates when relying on self-reported data on PHS utilization. This limitation could be mitigated in future studies utilizing the latest institutional survey data. Lastly, this study exclusively explored the impact of utilizing government-funded routine health examinations on the HRQoL of chronic disease patients. Given the ongoing expansion of PHS programs, future research endeavors could assess the impact of other programs and explore their influencing mechanisms on patients’ health outcomes and quality of life.

### Policy implications

To our knowledge, this study represents the first comprehensive exploration of the impact of community-based PHS on the HRQoL among Chinese middle-aged and older adults with chronic diseases. Building upon the aforementioned findings, we offer several implications for policymakers. Firstly, there is a need for the government to enhance the delivery and utilization of publicly funded PHS. This involves improving the accessibility of community-based health examination services by establishing more convenient locations and expanding service hours to accommodate diverse schedules. Additionally, implementing targeted health education and promotion campaigns is crucial to heighten health awareness about the advantages of regular health check-ups and PHS programs. Emphasis should be placed on the importance of early prevention and intervention, particularly in addressing the prevalence of multimorbidity.

Secondly, our findings highlight the significance of considering patients’ characteristics when assessing the effectiveness of community-based PHS among chronic disease patients. On the one hand, this study identifies a positive impact on HRQoL among those at an older age and with comorbidities, highlighting the urgent need for chronic disease management and financial protection support due to medical expenditure. Multimorbidity not only leads to higher mortality and poorer quality of life [57] but also imposes health-related financial and emotional stress on patients and their families [58]. Consequently, we recommend the government develop tailored health security programs for those with comorbidities, focusing on medication adherence and financial risk protection. Furthermore, there is a call for enhanced community-based caregiving and psychological support aimed at addressing the needs of older chronic disease patients. For middle-aged patients and those without obvious complications, it is advisable to develop tailored health promotion programs focusing on lifestyle modification, early symptom recognition, and health literacy from a young age.

On the other hand, given the greater health promotion effect of community-based PHS among urban patients compared with their rural counterparts, the government should intensify efforts to enhance the quality of rural community healthcare services. This involves strengthening and motivating the primary care workforce, integrating health service delivery, and consolidating rural-oriented financing arrangements [54]. We also recommend the development and implementation of community-based health education programs in rural areas to raise health literacy and awareness about chronic diseases. Utilizing mobile health technologies can bridge the urban-rural gap in healthcare access and empower patients to actively participate in their own care.

## Conclusions

Based on nationally representative survey data, this study focused on hypertensive and diabetic patients, exploring the impact of community-based PHS programs on the HRQoL in middle-aged and older adults with chronic diseases in China. Employing multivariate linear regression models with IVs and PSM, our findings revealed positive effects of PHS on both the physiological and psychological dimensions of quality of life among individuals with chronic diseases. Particularly noteworthy was the greater positive impact on mental well-being. Through the stratification of samples based on various characteristics, our study provided nuanced insights. We observed a positive effect of PHS on HRQoL across gender, age groups (with a notable impact on older patients), patients with comorbidities, and urban residents. These findings highlighted the potential benefits of tailored health interventions for specific demographic and health profile groups. The subsequent phase involves conducting additional research to investigate additional PHS projects and examining their impact mechanisms on patients’ health outcomes. We recommend enhancing the delivery and utilization of PHS, increasing health literacy and promoting the awareness of chronic disease prevention, implementing targeted health management programs for those with comorbidities, and improving the quality of community healthcare services, particularly in rural areas.

### Electronic supplementary material

Below is the link to the electronic supplementary material.

Supplementary Material 1

## Data Availability

The data source of this study was a publicly available database, the China Health and Retirement Longitudinal Study, which was hosted by the National Development Center of Peking University. The data are available at http://charls.pku.edu.cn/index.htm.

## Abbreviations

BP: Body Pain
CHARLS: China Health and Retirement Longitudinal Study
FFM: Functional Form Misspecification
GDP: Gross Domestic Product
GH: General Health
HbA1c: Glycated Hemoglobin
HRQoL: Health-related Quality of Life
IRB: Institutional Review Board
IV: Instrument Variable
MCS: Mental Component Summary
MH: Mental Health
PCS: Physical Component Summary
PF: Physical Function
PHS: Public Health Service
PHI: Private Health Insurance
PSM: Propensity Score Matching
RE: Role–emotion
RP: Role–physical
SF-36: 36-Item Short Form Health Survey
SF: Social Functioning
SHI: Social Health Insurance
2SLS: Two-stage Least Squares
UR: Utilization Rate
VT: Vitality
