# Supplementary material for: Effect of community-based public health service on health-related quality of life among middle-aged and older adults with chronic diseases in China
Source: BMC Public Health. 2024 Jul 30;24:2039. doi: 10.1186/s12889-024-19556-w (PMC11290236; doi:10.1186/s12889-024-19556-w)
Supplement: Supplementary file 1 — Supplementary Material 1 [file 12889_2024_19556_MOESM1_ESM.docx]

**Additional file**

**Table A1 Control variable test results before and after caliper matching among patients with hypertension or/and diabetes**

| Variable | Unmatched  /Matched | Mean | | | %reduct  \|bias\| | t-test | |
| --- | --- | --- | --- | --- | --- | --- | --- |
|  |  | Treated | Control | %bias |  | t | p>\|t\| |
| Age | U | 66.954 | 62.551 | 47.60 |  | 12.570 | <0.001 |
|  | M | 66.913 | 67.031 | -1.30 | 97.30 | -0.270 | 0.789 |
| Gender | U | 1.528 | 1.532 | -1.00 |  | -0.260 | 0.793 |
|  | M | 1.529 | 1.535 | -1.20 | -28.40 | -0.260 | 0.793 |
| Education: Primary school | U | 0.433 | 0.407 | 5.40 |  | 1.510 | 0.132 |
|  | M | 0.433 | 0.434 | -0.10 | 97.70 | -0.030 | 0.978 |
| Education: Middle school | U | 0.164 | 0.196 | -8.30 |  | -2.250 | 0.024 |
|  | M | 0.164 | 0.156 | 2.00 | 76.30 | 0.440 | 0.657 |
| Education: High school and above | U | 0.090 | 0.083 | 2.40 |  | 0.670 | 0.505 |
|  | M | 0.089 | 0.083 | 2.00 | 14.40 | 0.440 | 0.661 |
| Occupation: Agricultural work | U | 0.323 | 0.368 | -9.50 |  | -2.580 | 0.010 |
|  | M | 0.324 | 0.331 | -1.60 | 83.50 | -0.340 | 0.735 |
| Occupation: Non-agricultural work | U | 0.158 | 0.218 | -15.30 |  | -4.060 | <0.001 |
|  | M | 0.159 | 0.156 | 0.80 | 95.10 | 0.170 | 0.862 |
| Living with a partner/spouse | U | 0.796 | 0.769 | 6.50 |  | 1.770 | 0.077 |
|  | M | 0.797 | 0.792 | 1.10 | 83.10 | 0.240 | 0.810 |
| A child living nearby | U | 0.888 | 0.846 | 12.60 |  | 3.320 | 0.001 |
|  | M | 0.888 | 0.890 | -0.70 | 94.60 | -0.160 | 0.875 |
| Number of chronic diseases | U | 2.819 | 2.409 | 23.30 |  | 6.590 | <0.001 |
|  | M | 2.813 | 2.818 | -0.30 | 98.90 | -0.050 | 0.958 |
| Number of disabilities | U | 0.232 | 0.208 | 4.40 |  | 1.240 | 0.215 |
|  | M | 0.231 | 0.239 | -1.30 | 69.40 | -0.270 | 0.784 |
| SHI | U | 0.921 | 0.876 | 14.90 |  | 3.880 | <0.001 |
|  | M | 0.921 | 0.921 | 0.00 | 99.80 | 0.010 | 0.993 |
| PHI | U | 0.017 | 0.014 | 3.20 |  | 0.910 | 0.364 |
|  | M | 0.017 | 0.017 | 0.00 | 99.20 | -0.010 | 0.996 |
| Log household expenditure per capital | U | 7.347 | 7.262 | 9.90 |  | 2.810 | 0.005 |
|  | M | 7.345 | 7.327 | 2.10 | 78.70 | 0.450 | 0.653 |
| Urban | U | 0.455 | 0.339 | 23.90 |  | 6.720 | <0.001 |
|  | M | 0.454 | 0.449 | 1.00 | 95.80 | 0.210 | 0.832 |
| Log GDP per capital | U | 8.918 | 8.684 | 44.20 |  | 12.490 | <0.001 |
|  | M | 8.916 | 8.907 | 1.70 | 96.10 | 0.370 | 0.712 |

Note: SHI, social health insurance; PHI, private health insurance; GDP, Gross Domestic Product.

**Table A2 Control variable test results before and after 1:4 nearest-neighbor matching within a caliper among patients with hypertension or/and diabetes**

| Variable | Unmatched  /Matched | Mean | | | %reduct  \|bias\| | t-test | |
| --- | --- | --- | --- | --- | --- | --- | --- |
|  |  | Treated | Control | %bias |  | t | p>\|t\| |
| Age | U | 66.954 | 62.551 | 47.60 |  | 12.570 | <0.001 |
|  | M | 66.913 | 67.223 | -3.40 | 93.00 | -0.700 | 0.485 |
| Gender | U | 1.528 | 1.532 | -1.00 |  | -0.260 | 0.793 |
|  | M | 1.529 | 1.547 | -3.60 | -275.40 | -0.770 | 0.443 |
| Education: Primary school | U | 0.433 | 0.407 | 5.40 |  | 1.510 | 0.132 |
|  | M | 0.433 | 0.428 | 1.10 | 79.40 | 0.240 | 0.811 |
| Education: Middle school | U | 0.164 | 0.196 | -8.30 |  | -2.250 | 0.024 |
|  | M | 0.164 | 0.151 | 3.50 | 58.50 | 0.780 | 0.433 |
| Education: High school and above | U | 0.090 | 0.083 | 2.40 |  | 0.670 | 0.505 |
|  | M | 0.089 | 0.087 | 0.60 | 75.70 | 0.120 | 0.902 |
| Occupation: Agricultural work | U | 0.323 | 0.368 | -9.50 |  | -2.580 | 0.010 |
|  | M | 0.324 | 0.313 | 2.20 | 77.00 | 0.470 | 0.635 |
| Occupation: Non-agricultural work | U | 0.158 | 0.218 | -15.30 |  | -4.060 | <0.001 |
|  | M | 0.159 | 0.162 | -0.90 | 94.10 | -0.210 | 0.837 |
| Living with a partner/spouse | U | 0.796 | 0.769 | 6.50 |  | 1.770 | 0.077 |
|  | M | 0.797 | 0.787 | 2.50 | 62.20 | 0.540 | 0.592 |
| A child living nearby | U | 0.888 | 0.846 | 12.60 |  | 3.320 | 0.001 |
|  | M | 0.888 | 0.908 | -5.80 | 53.40 | -1.410 | 0.160 |
| Number of chronic diseases | U | 2.819 | 2.409 | 23.30 |  | 6.590 | <0.001 |
|  | M | 2.813 | 2.821 | -0.40 | 98.20 | -0.090 | 0.930 |
| Number of disabilities | U | 0.232 | 0.208 | 4.40 |  | 1.240 | 0.215 |
|  | M | 0.231 | 0.245 | -2.60 | 40.40 | -0.530 | 0.596 |
| SHI | U | 0.921 | 0.876 | 14.90 |  | 3.880 | <0.001 |
|  | M | 0.921 | 0.914 | 2.10 | 86.10 | 0.490 | 0.626 |
| PHI | U | 0.017 | 0.014 | 3.20 |  | 0.910 | 0.364 |
|  | M | 0.017 | 0.017 | 0.70 | 79.00 | 0.140 | 0.892 |
| Log household expenditure per capital | U | 7.347 | 7.262 | 9.90 |  | 2.810 | 0.005 |
|  | M | 7.345 | 7.346 | -0.10 | 99.30 | -0.010 | 0.989 |
| Urban | U | 0.455 | 0.339 | 23.90 |  | 6.720 | <0.001 |
|  | M | 0.454 | 0.448 | 1.10 | 95.30 | 0.240 | 0.812 |
| Log GDP per capital | U | 8.918 | 8.684 | 44.20 |  | 12.490 | <0.001 |
|  | M | 8.916 | 8.906 | 1.90 | 95.60 | 0.420 | 0.677 |

Note: SHI, social health insurance; PHI, private health insurance; GDP, Gross Domestic Product.

**Table A3 Control variable test results before and after caliper matching among patients with hypertension**

| Variable | Unmatched  /Matched | Mean | | | %reduct  \|bias\| | t-test | |
| --- | --- | --- | --- | --- | --- | --- | --- |
|  |  | Treated | Control | %bias |  | t | p>\|t\| |
| Age | U | 67.276 | 62.855 | 47.60 |  | 11.910 | <0.001 |
|  | M | 67.177 | 67.344 | -1.80 | 96.20 | -0.360 | 0.716 |
| Gender | U | 1.519 | 1.523 | -0.90 |  | -0.230 | 0.821 |
|  | M | 1.522 | 1.524 | -0.60 | 32.70 | -0.120 | 0.906 |
| Education: Primary school | U | 0.439 | 0.409 | 6.20 |  | 1.640 | 0.101 |
|  | M | 0.439 | 0.441 | -0.30 | 94.90 | -0.060 | 0.948 |
| Education: Middle school | U | 0.164 | 0.193 | -7.60 |  | -1.950 | 0.051 |
|  | M | 0.165 | 0.156 | 2.50 | 67.10 | 0.530 | 0.595 |
| Education: High school and above | U | 0.087 | 0.080 | 2.60 |  | 0.690 | 0.490 |
|  | M | 0.085 | 0.081 | 1.30 | 50.10 | 0.260 | 0.791 |
| Occupation: Agricultural work | U | 0.312 | 0.368 | -11.90 |  | -3.080 | 0.002 |
|  | M | 0.313 | 0.323 | -2.00 | 83.20 | -0.410 | 0.679 |
| Occupation: Non-agricultural work | U | 0.161 | 0.214 | -13.70 |  | -3.490 | <0.001 |
|  | M | 0.161 | 0.158 | 0.80 | 94.20 | 0.170 | 0.863 |
| Living with a partner/spouse | U | 0.792 | 0.765 | 6.50 |  | 1.690 | 0.092 |
|  | M | 0.793 | 0.786 | 1.70 | 73.80 | 0.350 | 0.723 |
| A child living nearby | U | 0.889 | 0.850 | 11.70 |  | 2.960 | 0.003 |
|  | M | 0.889 | 0.892 | -0.80 | 92.90 | -0.180 | 0.854 |
| Number of chronic diseases | U | 2.874 | 2.446 | 24.10 |  | 6.510 | <0.001 |
|  | M | 2.857 | 2.879 | -1.30 | 94.80 | -0.250 | 0.804 |
| Number of disabilities | U | 0.232 | 0.209 | 4.30 |  | 1.170 | 0.240 |
|  | M | 0.232 | 0.235 | -0.60 | 86.90 | -0.110 | 0.911 |
| SHI | U | 0.920 | 0.875 | 14.90 |  | 3.680 | <0.001 |
|  | M | 0.920 | 0.919 | 0.20 | 98.30 | 0.0600 | 0.956 |
| PHI | U | 0.017 | 0.013 | 2.70 |  | 0.730 | 0.468 |
|  | M | 0.017 | 0.016 | 0.70 | 74.70 | 0.130 | 0.895 |
| Log household expenditure per capital | U | 7.349 | 7.249 | 11.40 |  | 3.090 | 0.002 |
|  | M | 7.346 | 7.323 | 2.70 | 76.70 | 0.540 | 0.589 |
| Urban | U | 0.460 | 0.335 | 25.50 |  | 6.830 | <0.001 |
|  | M | 0.457 | 0.451 | 1.20 | 95.10 | 0.250 | 0.805 |
| Log GDP per capital | U | 8.917 | 8.681 | 44.90 |  | 12.030 | <0.001 |
|  | M | 8.915 | 8.904 | 2.20 | 95.10 | 0.450 | 0.652 |

Note: SHI, social health insurance; PHI, private health insurance; GDP, Gross Domestic Product.

**Table A4 Control variable test results before and after 1:4 nearest-neighbor matching within a caliper among patients with hypertension**

| Variable | Unmatched  /Matched | Mean | | | %reduct  \|bias\| | t-test | |
| --- | --- | --- | --- | --- | --- | --- | --- |
|  |  | Treated | Control | %bias |  | t | p>\|t\| |
| Age | U | 67.276 | 62.855 | 47.60 |  | 11.910 | <0.001 |
|  | M | 67.177 | 67.163 | 0.20 | 99.70 | 0.030 | 0.975 |
| Gender | U | 1.519 | 1.523 | -0.90 |  | -0.230 | 0.821 |
|  | M | 1.522 | 1.522 | -0.10 | 86.10 | -0.020 | 0.980 |
| Education: Primary school | U | 0.439 | 0.409 | 6.20 |  | 1.640 | 0.101 |
|  | M | 0.439 | 0.450 | -2.30 | 63.10 | -0.470 | 0.640 |
| Education: Middle school | U | 0.164 | 0.193 | -7.60 |  | -1.950 | 0.051 |
|  | M | 0.165 | 0.151 | 3.60 | 52.60 | 0.770 | 0.441 |
| Education: High school and above | U | 0.087 | 0.080 | 2.60 |  | 0.690 | 0.490 |
|  | M | 0.085 | 0.088 | -1.10 | 58.30 | -0.220 | 0.828 |
| Occupation: Agricultural work | U | 0.312 | 0.368 | -11.90 |  | -3.080 | 0.002 |
|  | M | 0.313 | 0.315 | -0.30 | 97.90 | -0.050 | 0.958 |
| Occupation: Non-agricultural work | U | 0.161 | 0.214 | -13.70 |  | -3.490 | <0.001 |
|  | M | 0.161 | 0.160 | 0.50 | 96.60 | 0.100 | 0.920 |
| Living with a partner/spouse | U | 0.792 | 0.765 | 6.50 |  | 1.690 | 0.092 |
|  | M | 0.793 | 0.791 | 0.60 | 91.10 | 0.120 | 0.904 |
| A child living nearby | U | 0.889 | 0.850 | 11.70 |  | 2.960 | 0.003 |
|  | M | 0.889 | 0.891 | -0.60 | 94.70 | -0.140 | 0.891 |
| Number of chronic diseases | U | 2.874 | 2.446 | 24.10 |  | 6.510 | <0.001 |
|  | M | 2.857 | 2.891 | -1.90 | 92 | -0.380 | 0.702 |
| Number of disabilities | U | 0.232 | 0.209 | 4.30 |  | 1.170 | 0.240 |
|  | M | 0.232 | 0.235 | -0.50 | 87.30 | -0.110 | 0.914 |
| SHI | U | 0.920 | 0.875 | 14.90 |  | 3.680 | <0.001 |
|  | M | 0.920 | 0.919 | 0.40 | 97.30 | 0.090 | 0.929 |
| PHI | U | 0.017 | 0.013 | 2.70 |  | 0.730 | 0.468 |
|  | M | 0.017 | 0.021 | -3.70 | -38.10 | -0.670 | 0.502 |
| Log household expenditure per capital | U | 7.349 | 7.249 | 11.40 |  | 3.090 | 0.002 |
|  | M | 7.346 | 7.346 | -0.01 | 99.70 | -0.010 | 0.995 |
| Urban | U | 0.460 | 0.335 | 25.50 |  | 6.830 | <0.001 |
|  | M | 0.457 | 0.456 | 0.20 | 99.30 | 0.040 | 0.971 |
| Log GDP per capital | U | 8.917 | 8.681 | 44.90 |  | 12.030 | <0.001 |
|  | M | 8.915 | 8.909 | 1.20 | 97.30 | 0.250 | 0.801 |

Note: SHI, social health insurance; PHI, private health insurance; GDP, Gross Domestic Product.

**Table A5 Control variable test results before and after caliper matching among patients with diabetes**

| Variable | Unmatched  /Matched | Mean | | | %reduct  \|bias\| | t-test | |
| --- | --- | --- | --- | --- | --- | --- | --- |
|  |  | Treated | Control | %bias |  | t | p>\|t\| |
| Age | U | 66.071 | 61.995 | 46.80 |  | 6.960 | <0.001 |
|  | M | 65.790 | 65.955 | -1.90 | 95.90 | -0.220 | 0.824 |
| Gender | U | 1.596 | 1.606 | -2.10 |  | -0.330 | 0.743 |
|  | M | 1.607 | 1.609 | -0.40 | 81.20 | -0.050 | 0.962 |
| Education: Primary school | U | 0.424 | 0.400 | 4.90 |  | 0.750 | 0.453 |
|  | M | 0.431 | 0.422 | 1.80 | 62.50 | 0.220 | 0.828 |
| Education: Middle school | U | 0.158 | 0.193 | -9.30 |  | -1.390 | 0.163 |
|  | M | 0.162 | 0.158 | 1.00 | 89.10 | 0.130 | 0.900 |
| Education: High school and above | U | 0.118 | 0.096 | 7.20 |  | 1.150 | 0.250 |
|  | M | 0.103 | 0.104 | -0.10 | 98.90 | -0.010 | 0.992 |
| Occupation: Agricultural work | U | 0.283 | 0.338 | -11.80 |  | -1.800 | 0.072 |
|  | M | 0.290 | 0.299 | -2.10 | 82.40 | -0.250 | 0.799 |
| Occupation: Non-agricultural work | U | 0.104 | 0.207 | -28.50 |  | -4.080 | <0.001 |
|  | M | 0.107 | 0.099 | 2.20 | 92.30 | 0.310 | 0.755 |
| Living with a partner/spouse | U | 0.778 | 0.799 | -5.20 |  | -0.810 | 0.418 |
|  | M | 0.783 | 0.774 | 2.10 | 59.70 | 0.250 | 0.805 |
| A child living nearby | U | 0.892 | 0.838 | 15.80 |  | 2.320 | 0.020 |
|  | M | 0.890 | 0.893 | -1.10 | 93 | -0.150 | 0.884 |
| Number of chronic diseases | U | 3.465 | 2.927 | 28.20 |  | 4.360 | <0.001 |
|  | M | 3.407 | 3.433 | -1.40 | 95.20 | -0.160 | 0.873 |
| Number of disabilities | U | 0.266 | 0.242 | 4.00 |  | 0.640 | 0.520 |
|  | M | 0.266 | 0.275 | -1.60 | 60.80 | -0.190 | 0.852 |
| SHI | U | 0.916 | 0.886 | 10.00 |  | 1.480 | 0.140 |
|  | M | 0.914 | 0.919 | -1.70 | 82.80 | -0.220 | 0.824 |
| PHI | U | 0.014 | 0.016 | -2.00 |  | -0.310 | 0.760 |
|  | M | 0.014 | 0.013 | 0.30 | 85 | 0.040 | 0.970 |
| Log household expenditure per capital | U | 7.410 | 7.329 | 10.30 |  | 1.610 | 0.107 |
|  | M | 7.410 | 7.365 | 5.60 | 45.40 | 0.660 | 0.508 |
| Urban | U | 0.512 | 0.390 | 24.60 |  | 3.820 | <0.001 |
|  | M | 0.500 | 0.493 | 1.50 | 93.90 | 0.180 | 0.859 |
| Log GDP per capital | U | 8.932 | 8.728 | 38.40 |  | 6.100 | <0.001 |
|  | M | 8.917 | 8.897 | 3.90 | 89.90 | 0.480 | 0.634 |

Note: SHI, social health insurance; PHI, private health insurance; GDP, Gross Domestic Product.

**Table A6 Control variable test results before and after 1:4 nearest-neighbor matching among patients with diabetes**

| Variable | Unmatched  /Matched | Mean | | | %reduct  \|bias\| | t-test | |
| --- | --- | --- | --- | --- | --- | --- | --- |
|  |  | Treated | Control | %bias |  | t | p>\|t\| |
| Age | U | 66.071 | 61.995 | 46.80 |  | 6.960 | <0.001 |
|  | M | 65.790 | 65.733 | 0.70 | 98.60 | 0.080 | 0.938 |
| Gender | U | 1.596 | 1.606 | -2.10 |  | -0.330 | 0.743 |
|  | M | 1.607 | 1.620 | -2.60 | -24.30 | -0.320 | 0.750 |
| Education: Primary school | U | 0.424 | 0.400 | 4.90 |  | 0.750 | 0.453 |
|  | M | 0.431 | 0.418 | 2.60 | 45.90 | 0.310 | 0.753 |
| Education: Middle school | U | 0.158 | 0.193 | -9.30 |  | -1.390 | 0.163 |
|  | M | 0.162 | 0.161 | 0.20 | 97.60 | 0.030 | 0.978 |
| Education: High school and above | U | 0.118 | 0.096 | 7.20 |  | 1.150 | 0.250 |
|  | M | 0.103 | 0.108 | -1.40 | 80.70 | -0.170 | 0.866 |
| Occupation: Agricultural work | U | 0.283 | 0.338 | -11.80 |  | -1.800 | 0.072 |
|  | M | 0.290 | 0.284 | 1.30 | 89 | 0.160 | 0.873 |
| Occupation: Non-agricultural work | U | 0.104 | 0.207 | -28.50 |  | -4.080 | <0.001 |
|  | M | 0.107 | 0.094 | 3.60 | 87.40 | 0.520 | 0.605 |
| Living with a partner/spouse | U | 0.778 | 0.799 | -5.20 |  | -0.810 | 0.418 |
|  | M | 0.783 | 0.777 | 1.50 | 71.60 | 0.180 | 0.861 |
| A child living nearby | U | 0.892 | 0.838 | 15.80 |  | 2.320 | 0.020 |
|  | M | 0.890 | 0.884 | 1.50 | 90.40 | 0.200 | 0.844 |
| Number of chronic diseases | U | 3.465 | 2.927 | 28.20 |  | 4.360 | <0.001 |
|  | M | 3.407 | 3.389 | 1.00 | 96.60 | 0.110 | 0.912 |
| Number of disabilities | U | 0.266 | 0.242 | 4.00 |  | 0.640 | 0.520 |
|  | M | 0.266 | 0.248 | 2.900 | 28 | 0.360 | 0.720 |
| SHI | U | 0.916 | 0.886 | 10.00 |  | 1.480 | 0.140 |
|  | M | 0.914 | 0.918 | -1.40 | 85.50 | -0.190 | 0.852 |
| PHI | U | 0.014 | 0.016 | -2.00 |  | -0.310 | 0.760 |
|  | M | 0.014 | 0.009 | 3.60 | -76.30 | 0.480 | 0.629 |
| Log household expenditure per capital | U | 7.410 | 7.329 | 10.30 |  | 1.610 | 0.107 |
|  | M | 7.410 | 7.401 | 1.10 | 88.90 | 0.130 | 0.893 |
| Urban | U | 0.512 | 0.390 | 24.60 |  | 3.820 | <0.001 |
|  | M | 0.500 | 0.509 | -1.90 | 92.20 | -0.230 | 0.820 |
| Log GDP per capital | U | 8.932 | 8.728 | 38.40 |  | 6.100 | <0.001 |
|  | M | 8.917 | 8.911 | 1.20 | 96.80 | 0.150 | 0.880 |

Note: SHI, social health insurance; PHI, private health insurance; GDP, Gross Domestic Product.

**Figure A1 Common value range of propensity score using caliper matching among patients with hypertension or/and diabetes**

**Figure A2 Common value range of propensity score using 1:4 nearest-neighbor matching within caliper among patients with hypertension or/and diabetes**

**Figure A3 Common value range of propensity score using caliper matching among patients with hypertension**

**Figure A4 Common value range of propensity score using 1:4 nearest-neighbor matching within caliper among patients with hypertension**

**Figure A5 Common value range of propensity score using caliper matching among patients with diabetes**

**Figure A6 Common value range of propensity score using 1:4 nearest-neighbor matching within caliper among patients with diabetes**

(a) before matching (b) after matching

**Figure A7 Kernel density distribution between treatment and control groups using caliper matching among patients with hypertension or/and diabetes**

(a) before matching (b) after matching

**Figure A8 Kernel density distribution between treatment and control groups using 1:4 nearest-neighbor matching within caliper among patients with hypertension or/and diabetes**

(a) before matching (b) after matching

**Figure A9 Kernel density distribution between treatment and control groups using caliper matching among patients with hypertension**

(a) before matching (b) after matching

**Figure A10 Kernel density distribution between treatment and control groups using 1:4 nearest-neighbor matching within caliper among patients with hypertension**

(a) before matching (b) after matching

**Figure A11 Kernel density distribution between treatment and control groups using caliper matching among patients with diabetes**

(a) before matching (b) after matching

**Figure A12 Kernel density distribution between treatment and control groups using 1:4 nearest-neighbor matching within caliper among patients with diabetes**
